# Production optimization of cyanophycinase ChpE_al _from *Pseudomonas alcaligenes *DIP1

**DOI:** 10.1186/2191-0855-1-38

**Published:** 2011-11-07

**Authors:** Ahmed Sallam, Dimitar Kalkandzhiev, Alexander Steinbüchel

**Affiliations:** 1 Institut für Molekulare Mikrobiologie und Biotechnologie, Westfälische Wilhelms-Universität Münster, D-48149 Münster, Germany

**Keywords:** Cyanophycinase, Cyanophycin degradation, *Pseudomonas alcaligenes*

## Abstract

*Pseudomonas alcaligenes *DIP1 produces an extracellular cyanophycinase (CphE_al_). The corresponding gene (*cphE_al_*) was identified from subclones of a genomic DNA gene library by heterologously expressing the functionally active enzyme in *Escherichia coli*. The nucleotide sequence of the gene (1260 base pairs) was determined indicating a theoretical mass of 43.6 kDa (mature CphE_al_) plus a leader peptide of 2,6 kDa which corresponds well to the apparent molecular mass of 45 kDa as revealed by SDS-PAGE. The enzyme exhibited a high sequence identity of 91% with the extracellular cyanophycinase from *P. anguilliseptica *strain BI and carried an N-terminal Sec secretion signal peptide. Analysis of the amino acid sequence of *cph*E revealed a putative catalytic triad consisting of the serine motif GXSXG plus a histidine and a glutamate residue, suggesting a catalytic mechanism similar to serine-type proteases. The cyanophycinase (CphE_al_) was heterologously produced in two different *E. coli *strains (Top10 and BL21(DE3)) from two plasmid vectors (pBBR1MCS-4 and pET-23a(+)). The signal peptide of CphE_al _was cleaved in *E. coli*, suggesting active export of the protein at least to the periplasm. Substantial enzyme activity was also present in the culture supernatants. The extracellular cyanophycinase activities in *E. coli *were higher than activities in the wild type *P. alcaligenes *DIP1 in complex LB medium. Highest extracellular enzyme production was achieved with *E. coli *BL21(DE3) expressing CphE_al _from pBBR1MCS-4. Using M9 minimal medium was less effective, but the relatively low cost of mineral salt media makes these results important for the industrial-scale production of dipeptides from cyanophycin.

## Introduction

Cyanophycin (cyanophycin granule polypeptide, CGP) is a naturally occurring poly(amino acid) that was first observed in cyanobacteria (Borzi 1887). It is accumulated in the early stationary growth phase (Mackerras et al. 1990; Liotenberg et al. 1996) and functions as storage compound for nitrogen, carbon, and energy (Elbahloul et al. 2005; Füser and Steinbüchel 2007). CGP consists of a poly(aspartic acid) backbone with arginine moieties linked to the β-carboxyl group of each aspartic acid by its α-amino group to form multi-L-arginyl-poly(L-aspartic acid) (Simon and Weathers 1976; Oppermann-Sanio and Steinbüchel 2003). CGP is accumulated intracellularly in the form of membraneless granules and is degraded by cells when growth is resumed (Allen and Weathers 1980).

Most genera of cyanobacteria (Simon 1987; Allen 1988; Mackerras et al. 1990; Liotenberg et al. 1996; Wingard et al. 2002) and also some heterotrophic bacteria (Krehenbrink et al. 2002; Ziegler et al. 2002; Füser and Steinbüchel 2007) harbor a cyanophycin synthetase gene (*cphA*) and are able to synthesize CGP. Although CGP is generally insoluble at neutral pH as well as at physiological ionic strength (Allen et al. 1980), a water-soluble form of CGP was also synthesized in recombinant *Escherichia coli *and yeast (Ziegler et al. 2002; Steinle et al. 2009; 2010). CGP from cyanobacteria exhibits a molecular mass of 25-100 kDa (Simon 1976), while CGP from heterotrophic bacteria and recombinant strains is smaller and much less polydisperse (25-30 kDa) (Ziegler et al. 1998; Aboulmagd et al. 2001; Krehenbrink et al. 2002).

CGP is resistant against hydrolytic cleavage by most proteases and arginase (Berg 2003) but is degraded by dedicated intracellular or extracellular cyanophycinases (CphB and CphE, respectively). The first reported CGPase, an intracellular CphB, was described by Gupta and Carr (1981) as a CGP-specific serine-type exopeptidase with an α-cleavage mechanism. Later, several aerobic and anaerobic bacteria capable of degrading CGP by extracellular CphEs were isolated. The extracellular CGPases CphE_Pa _from *Pseudomonas anguilliseptica *BI, CphE_Bm _from *Bacillus megaterium *BAC19, and CphE_al _from the facultatively anaerobic *Pseudomonas alcaligenes *DIP1 were subsequently purified and characterized (Obst et al. 2002; 2004; Sallam et al. 2009). Similar to CphB, all three CphEs were identified as CGP-specific serine-type proteases that produced Asp-Arg dipeptides as degradation products.

Until recently, no practical applications for CGP itself or for the constituent dipeptides were known. In contrast, economically important applications are widely established for poly(aspartic acid) as a substitute for non-biodegradable polyacrylates (Schwamborn 1998) or as an additive in the paper, paint and oil industries (Joentgen et al. 2003). Biomedical applications have been also described for poly(aspartic acid) (Yokoyama et al. 1990; Leopold and Friend 1995). Besides being a potential natural source for poly(aspartic acid), biomedical applications for dipeptides derived from CGP were recently proposed (Sallam and Steinbüchel 2010); these applications were based on two scientific findings: i) the astonishingly widespread occurrence of CGP-degrading bacteria in the digestive tracts of various vertebrates which indicates that CGP is probably degradable in these habitats, and ii) the higher bioavailability of ingested dipeptides compared to free amino acids. Thus, CGP dipeptides were proposed as potential natural additives for the pharmaceutical and food industries (Sallam and Steinbüchel 2010).

Several cyanobacterial *cphA *genes were heterologously expressed in *E. coli*, *Corynebacterium glutamicum*, *Ralstonia eutropha*, and *Pseudomonas putida *and were applied in the large scale production of CGP (Elbahloul et al 2005). CGP was also produced in tobacco and potato plants (Neumann et al. 2005) and in yeast (Steinle et al. 2009; 2010). To approach an economical large-scale production of CGP dipeptides, a technical process for their large scale degradation was developed (Sallam et al. 2009). The latter study describes a production process which depends on the use of CphE_al _from *Pseudomonas alcaligenes *DIP1 for CGP degradation and describes also the biochemical characteristics of this extracellular CGPase. However, this process still relied on the production of the enzyme from its native source. In the current study, we report on the heterologous expression of CphE_al _from *P. alcaligenes *DIP1 in *E. coli *strains. The aim of this work was to achieve a more efficient enzyme production than in *Pseudomonas alcaligenes *DIP1 and thereby enhance the overall productivity of the previously described process.

## Materials and methods

### Media

The previously described SM medium containing 2 g/l citrate as carbon source (Sallam et al. 2009) was used for cultivating *P. alcaligenes *DIP1 for the production of CphE_al_. The clarity of this mineral salt medium allows an efficient monitoring of turbidity changes during cultivation. This in turn provides an indicator for the release of CphE_al _by observation of the degradation of the insoluble inducer (CGP). CGP-overlay agar plates were prepared using a sterile suspension of CGP in 1.2% (wt/vol) Bacto agar, which was poured as thin layers onto SM agar plates (with 1 g/l yeast extract) (Sallam and Steinbüchel 2008).

To cultivate *E. coli *Top10 and *E. coli *BL21(DE3) for the heterologous production of CphE_al_, mineral salt medium (M9) containing 2% (vol/vol) glycerol or lysogeny broth (LB) medium (Sambrook et al. 1989) was used. Additionally, LB medium was also used for the maintenance of all used strains.

### Strains and plasmids

The wild type *P. alcaligenes *DIP1 (DSM 21533; Sallam et al. 2009) was used as control for CphE_al _production and also as donor for the *cphE_al _*gene. *E. coli *strains Top10 and BL21(DE3) (Novagen) were used in combination with the plasmid vectors pBBR1MCS-4 (Kovach et al. 1995) and pET-23a(+) (Novagen) for the heterologous production of the enzyme.

### Isolation, manipulation and analysis of DNA

MiniPrep Kit and DNeasy kit (Qiagen) were used for the isolation of plasmid DNA and total genomic DNA, respectively. To construct the *P. alcaligenes *DIP1 DNA library, genomic DNA of *P. alcaligenes *DIP1 was digested with *Pst*I and ligated into the vector pBluescriptSK^- ^(Stratagene). *E. coli *Mach1 (Invitrogen) was used as the library recipient. *E. coli *strain Top10 (Invitrogen) and plasmid pBBR1MCS-4 (Kovach et al. 1995) were used to subclone and sequence DNA fragments containing *cphE_al_*. Sequence analysis was performed by Seqlab (Göttingen, Germany). Nucleic acid sequence data and deduced amino acid sequences were analyzed using BLAST (Altschul et al. 1990), Genamics Expression 1.1 and SignalP 3.0 (Bendtsen et al. 2004) server. The DNA sequence was aligned with published sequences from representative *Pseudomonas *species from the National Center for Biotechnology Information (NCBI) data base. After identification of the *cphE *sequence, oligonucleotide PCR primers were designed for the amplification of the gene and for the construction of plasmids pBBR1MCS-4::*cphE_al _*and pET-23a(+)::*cphE_al_*. Primer P1 (CCTTAGGATCCAATAATTGATCCACGCGTTTTCGC) was designed to bind 66 bp upstream of the *cphE *coding sequence and to introduce a *Bam*HI restriction site (underlined). Primer P2 (GGATGAATTCTTATTTCAGTCGGGACGGAAGTCGG) was chosen to bind directly downstream of the *cphE *stop codon and to introduce an *Eco*RI restriction site (underlined).

### Concentration and screening of cyanophycinase

Proteins in culture supernatant samples were concentrated (20-fold) and desalted using 10-kDa-molecular weight cut-off Vivaspin tubes (Vivascience AG, Hannover, Germany) or, for volumes of up to 500 ml, an Amicon chamber (Amicon, Beverly, MA) with 10-kDa cut-off membranes (Millipore Corporation, Bedford, MA). ChpE_al _was purified by the previously described substrate affinity procedure which depends on the strong affinity of the enzyme to CGP (Sallam et al. 2009). Active CphE_al _fractions were identified by the appearance of degradation halos around 3 μl aliquots spotted on CGP-overlay plates after 5 to 40 min incubation at 37°C. For quantitative determination of the enzyme in culture samples, the previously described photometric assay for CphE_al _was applied (Sallam et al. 2009).

### Analytical techniques

Bacterial growth and CGP degradation were monitored by measuring changes in turbidity in Erlenmeyer flasks, using a Klett photometer (Manostat Corporation, NY) or a photometer (600 nm wavelength; Ultropec 2000 III photometer, Pharmacia Biotech, Uppsala, Sweden). Sodium dodecyl sulfate-polyacrylamide gel electrophoresis (SDS-PAGE) was performed in 11.5% (wt/vol) gels according to the method of Laemmli (1970). Proteins and CGP were visualized by Coomassie blue staining (Weber and Osborn 1969). Free amino acids and dipeptides were detected by HPLC (Kontron Instruments, Neufahrn, Germany) after precolumn derivatization with ortho-phthaldialdehyde (OPA) as described before (Aboulmagd et al. 2000; Sallam and Steinbüchel 2008). CGP samples were subjected to acid hydrolysis (6 N HCl, 95°C, overnight) before amino acid analysis.

## Results

### Cloning and analysis of *cphE_al _*from *P. alcaligenes *DIP1

Partial digestion of genomic DNA isolated from *P. alcaligenes *DIP1 with the restriction endonuclease *Pst*I yielded fragments with a broad size range that were subsequently ligated into the plasmid vector pBluescriptSK^-^. After transformation of the gene library into *E. coli *Mach1, about 3000 ampicillin-resistant clones were obtained and screened for their ability to degrade CGP on CGP-overlay agar plates. After 48 h of incubation at 37°C, a single colony was detected that was surrounded by a small degradation halo. The slow and weak degradation around the colonies of this clone may indicate poor gene expression or absence of active secretion of the enzyme. Sequence analysis of the plasmid from this clone revealed a 3.6-kbp genomic fragment.

### Molecular characterization of the CGPase gene (*cphE_al_*) from *P. alcaligenes *DIP1

**Figure 1:**
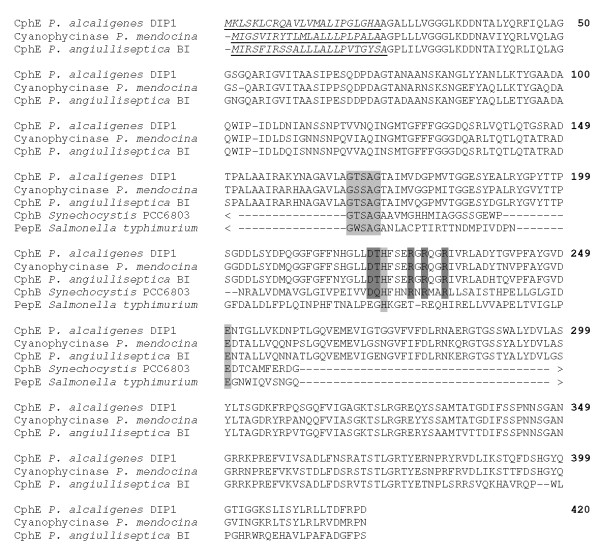
**Alignment of the deduced amino acid sequence of CphE_al _with other known and predicted cyanophycinases and PepE**. The potential signal peptides of the extracellular CGPases are underlined. Only the residues in the vicinity of the catalytic site are shown for CphB and PepE. The proposed catalytic residues (GXSXG, H and E) are shaded light gray. Residues proposed to be involved in substrate binding (Law et al. 2009) are shaded dark gray. For sequence alignment, the CLUSTAW was used. CphE_al _from *P. alcaligenes *DIP1; Cyanophycinase from *P. mendocina*; CphE_pa _from *P. anguilliseptica *BI; Cyanophycinase from *Synechocystis *PCC6803; PepE from *Salmonella typhimurium*. Numbering for the CphE_al _protein is given at the end of the line.

The N-terminus of CphE_al _was identified in antilinear orientation to the *lacZ *promoter of the vector by DNA sequence analysis of the cloned fragment; it was therefore concluded that the gene was expressed under the control of its own promoter. A predicted Sec secretion signal peptide of 24 amino acids with a signal peptidase cleavage site (HA-AG) was found in the deduced amino acid sequence preceding the N-terminus of the mature enzyme (Figure 1). A purine-rich sequence (GGAGAA) was detected 7-12 base pairs upstream of the methionine codon (ATG) of the leader peptide sequence, indicating a potential ribosome binding site in the corresponding mRNA transcript of the gene. An open reading frame of 1260 bp with TGA as stop codon was identified, corresponding to a theoretical protein mass of 43.6 kDa for the mature CphE_al _protein if the mass of the leader peptide (2,6 kDa) is not considered. This size corresponds well with the apparent molecular mass of the enzyme (subunit) that was determined by SDS-PAGE (45 kDa) (Sallam et al. 2009). The nucleotide sequence was submitted to GenBank under accession number [JN620418]

Heterologous production of CphE_al _by *E. coli *strains Top10 and BL21(DE3) with plasmid vectors pBBR1MCS-4 and pET-23a(+).

**Figure 2:**
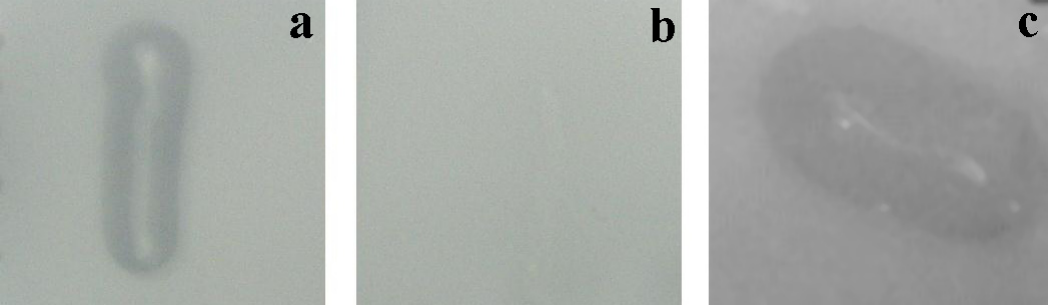
**Degradation halo formation around CGPase producing *E. coli *strains on CGP-overlay agar plates**. a: *E. coli *strain Top10 expressing CphE_al _from plasmid pBBR1MCS-4; b: *E. coli *strain Top10 expressing CphE_al _from plasmid pBluescriptSK^-^; c: *E. coli *strain BL21(DE3) expressing CphE_al _from plasmid pET-23a(+).

The fragment with *cphE *gene was cloned into the vector pBBR1MCS-4, and the enzyme expression in *E. coli *strain Top10 was confirmed on a CGP-overlay agar plate. The comparison between the two expression vectors (pBluescriptSK^-^::*cphE(PstI) *and pBBR1MCS-4::*cphE(PstI)*) showed much larger degradation halos with pBBR1MCS-4::*cphE(PstI) *after overnight incubation of CGP-overlay plates at 37°C (Figure 2).

To further improve the production of this cyanophycinase, the gene was amplified by PCR using primers P1 and P2, and the PCR product was cloned into the expression vector pET-23a(+) and expressed in *E. coli *strain BL21(DE3). Subsequent incubation on CGP-overlay agar plates showed good enzyme expression (Figure 2) as in the case of plasmid pBBR1MCS-4::*cphE(PstI) *and *E. coli *strain Top10. The expression occurred in the absence of an inducer, suggesting that the native *cphE *promoter is functional in *E. coli*. To identify the optimal combination of strain, plasmid and culture media, the fragment was also cloned into pBBR1MCS-4, and both plasmids were transferred to both strains. The production of CphE_al _was assayed in complex LB medium and minimal M9 medium with 2% (vol/vol) glycerol. For increased enzyme production with pET-23a(+) in *E. coli *BL21(DE3) 0.6 mM IPTG was added to the media.

**Figure 3:**
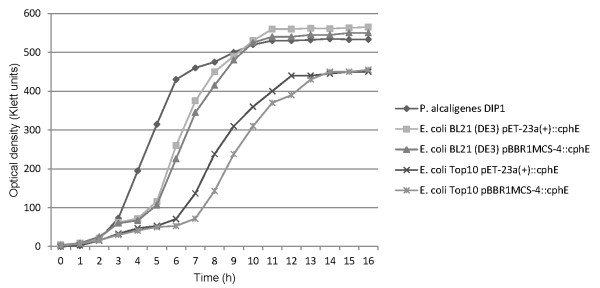
**Growth of the cyanophycinase producers in complex LB medium**. The cells were grown in 100 ml medium in 1000-ml Erlenmeyer flasks.

**Figure 4:**
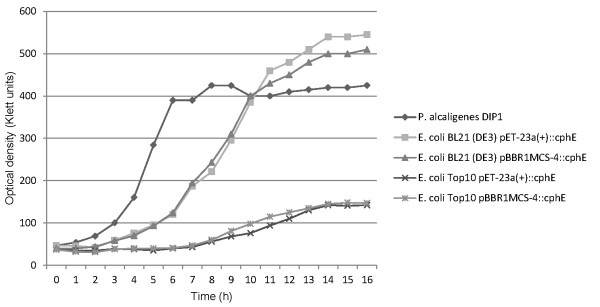
**Growth of the cyanophycinase producers in minimal M9 medium**. The cells were grown in 100 ml medium in 1000-ml Erlenmeyer flasks.

The growth of all strains, including *P. alcaligenes *DIP1, on LB complex medium (Figure 3) was very similar. The *P. alcaligenes *DIP1 wild type and *E. coli *strain BL21(DE3) with either plasmid reached a final optical density of 550 Klett units, while *E. coli *strain Top10 grew to an optical density of 450 Klett units. In mineral salt medium (M9) containing 2% glycerol as a carbon source *E. coli *BL21(DE3) with either plasmid reached an optical density of about 500 Klett units after 14 h. Enzyme production from pET-23a(+) was induced after 9 h at a cell density of 300 Klett units. *E. coli *Top10 reached an optical density of only 150 Klett units after 14 h in M9 medium (Figure 4).

**Figure 5:**
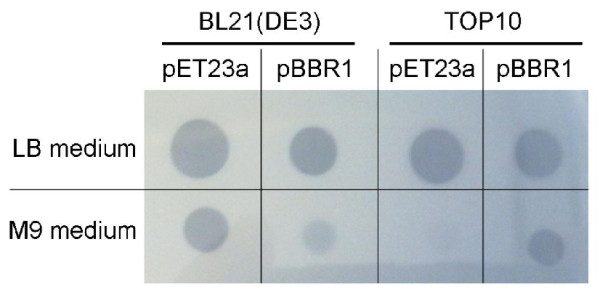
**CGPase activity in culture supernatants from *E. coli *strains Top10 and BL21(DE3) expressing CphE_al _from the plasmids pBBR1MCS-4 and pET-23a(+), assayed on CGP-overlay agar plate**.

The culture supernatants from all strains were concentrated 20-fold and tested for enzyme activity on CGP-overlay agar plates. Three microliters of the concentrated culture supernatants were spotted on plates and incubated for 1 h at 37°C (Figure 5). High CGPase activities were present in the culture supernatants of all strains grown in LB medium. Although the clear zones produced with plasmid pBBR1MCS-4::*cphE_al _*in LB appeared slightly smaller than with pET-23a(+)::*cphE_al _*for both strains, the activities were nevertheless high. The cyanophycinase activity produced by M9 cultures was much lower than that produced in LB medium. Although *E. coli *strain BL21(DE3) grew to similar cell densities in both cultures, the CGPase activities were much lower in M9 medium. *E. coli *Top10 pBBR1MCS-4::*cphE_al _*exhibited relatively high enzyme activities considering the low cell density achieved in M9 medium, but the overall activity was still much lower than that attained in LB medium. *E. coli *Top10 pET-23a(+)::*cphE_al_*, grown in M9 medium had almost no detectable enzyme activity.

**Figure 6:**
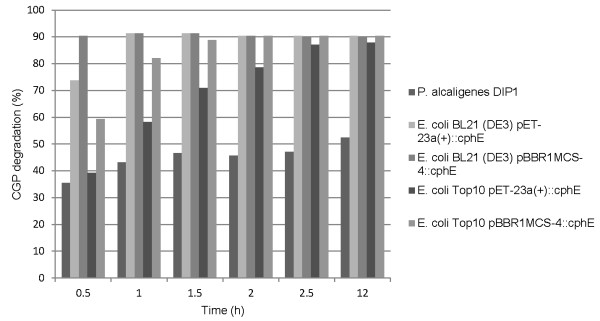
**CGP degradation by concentrated supernatant samples from cultures of *P. alcaligenes *DIP1, *E. coli *BL21(DE3) pET-23a(+)::*cphE*_al_, *E. coli *BL21(DE3) pBBR1MCS-4::*cphE*_al_, *E. coli *Top10 pET-23a(+)::*cphE*_al_, *E. coli *Top10 (DE3) pBBR1MCS-4::*cphE*_al _after induction**. CGP degradation was determined photometrically and was used as an indicator of the CphE_al _concentration.

As the CGP-overlay agar assay can only give a crude estimation of enzyme activity, CGPase activity was also determined using a photometric assay (Figure 6) by measuring the decrease in OD_600 _of a CGP suspension (100 mg/l) caused by the addition of 10 μl crude culture supernatant to 5 ml of suspension. CphE_al _from *E. coli *BL21(DE3) pBBR1MCS-4::*cphE_al _*degraded about 90% of CGP within 30 min. Within the same time, CphE_al _from *E. coli *BL21(DE3) pET-23a(+)::*cphE_al _*degraded about 70% of CGP, CphE_al _from *E. coli *Top10 pBBR1MCS-4::*cphE_al _*about 60%, and CphE_al _from *E. coli *Top10 pET-23a(+) only about 35%. After 2.5 hours all heterologously produced enzymes had degraded 90% of CGP, while the amount of enzyme produced by the wild type had only degraded about 50% of CGP.

## Discussion

Several extracellular cyanophycinases were identified in the last few years, and various applications have been proposed for the dipeptides produced by the action of these enzymes on CGP (Sallam and Steinbüchel 2010). A process for the production of dipeptides from CGP based on the cyanophycinase from *P. alcaligenes *strain DIP1 was described in previous studies (Sallam et al. 2009), but the identity of the cyanophycinase gene remained unknown. This study identified the cyanophycinase gene of this strain and optimized the production of the enzyme in recombinant *E. coli *strains.

The molecular characterization of the *cphE_al _*gene revealed a DNA sequence that encodes a protein with a similarity of 91% to the extracellular CGPase (CphE_pa_) from *P. anguilliseptica *BI. Both enzymes exhibit almost identical molecular masses, with 45 kDa for CphE_al _from *P. alcaligenes *DIP1 and 43 kDa for CphE_pa _from *P. anguilliseptica *BI. By comparison, the intracellular CGPase from *Synechocystis *sp. PCC6803 (CphB) possesses an apparent molecular mass of only 27 kDa (Richter et al. 1999). The known or predicted extracellular CGPases were shown to be more closely related to each other than to any known CphB proteins. However, all CGPases shared common features.

In CphE_al _from *P. alcaligenes *DIP1, the amino acids Ser^171^, His^224 ^and Glu^251 ^may constitute the catalytic active site residues responsible for the hydrolytic cleavage of the α-amide bonds in the polymer backbone. Accordingly, the catalytic mechanism is suggested to be that of a serine protease as previously proposed for intracellular CphB (Law et al. 2009). This finding is in good agreement with the confirmed sensitivity of CphE_al _toward serine protease inhibitors (Sallam et al. 2009). In contrast to the characteristic aspartic acid residue of most serine proteases, the catalytic triad is replaced by glutamic acid in CphE_al_. The same amino acid replacement was observed in the intracellular CGPase of *Synechocystis *sp. PCC6803 and other cyanobacteria (Richter et al. 1999) and in the predicted sequence of the extracellular cyanophycinase from *P. anguilliseptica *BI (Obst et al. 2006). The hypothetical cyanophycinase from *Pseudomonas mendocina *also showed the same replacement of Asp with Glu at this position.

Intracellular CphB exhibit structural similarity to the aspartyl-dipeptidase PepE from *Salmonella typhimurium *(Carter and Miller 1984; Miller 1998; Law et al. 2009). In this structure, amino acid residues Gln^101^, Asp^172^, Gln^173^, Arg^178^, Arg^180 ^and Arg^183 ^were shown to be critical for CGPase activity and for the formation of a conserved binding pocket adjacent to the catalytic Ser^132^. With the exception of Gln^173^, these residues are also conserved in CphE (Gln^136^, Asp^222^, Arg^228^, Arg^230 ^and Arg^233^). A threonine residue (Thr^223^) replaces Gln^173 ^of CphB in CphE_al_. The proposed role of Gln^173 ^is the formation of a hydrogen bond with the catalytic histidine residue, thereby promoting the correct orientation and tautomeric state of the imidazole group (Law et al. 2009). The hydroxyl group of Thr^223 ^may similarly be involved in hydrogen bonding to the catalytic histidine His^224 ^in CphE.

The presence of a potential cleavable N-terminal secretion signal peptide in amino acid sequence deduced from *cph*E suggested that in *P. alcaligenes *DIP1 CphE_al _is exported to the periplasm via the Sec secretion system before it is further secreted into the extracellular medium. As the export of a protein from the periplasm to the extracellular space is usually achieved via Type II protein secretion systems, it is hypothesized that such a system may be responsible for the secretion of CphE in the parental strain. The observed cleavage of the signal peptide of recombinant CphE_al _produced in *E. coli *suggested that CphE_al _is also exported to at least the periplasm in *E. coli*. The extracellular CGP degradation observed in recombinant *E. coli *was somewhat surprising as *E. coli *lacks a functional Type II protein secretion system. However, the observed strong and rapid extracellular activity of CphE, in comparison to that shown by wild type, recommends the presence of an as yet unidentified secretion system rather than being due to leakage from the periplasm.

CphE was active when expressed heterologously in *E. coli *Mach1 pBluescriptSK^-^::*cphE_al _*but was not efficiently produced in this system. Cloning the *cphE_al _*gene into the plasmid pBBR1MCS-4 and expressing the gene in *E. coli *Top10 or BL21(DE3) strongly increased extracellular enzyme production. Expression of the gene from plasmid pET-23a(+) also resulted in high levels of extracellular enzyme production. Although *E. coli *Top10 lacks the T7 polymerase required for efficient transcription from pET-23a(+), large amounts of enzyme were produced when this strain was grown in complex LB medium, suggesting that the native *cphE *promoter is recognized and induced in *E. coli *under the conditions present in complex growth medium, clearly indicating the need for artificial induction. Nevertheless, *E. coli *BL21(DE3) was found to be superior to *E. coli *Top10 in terms of total extracellular enzyme production with either plasmid. The highest levels of extracellular enzyme production were achieved with *E. coli *BL21(DE3) expressing *cph*E from pBBR1MCS-4 in LB medium. It has to be emphasized that all *E. coli *systems were superior to the wild type (*P. alcaligenes *DIP1) when grown in complex LB medium. Using M9 minimal medium with glycerol as a carbon source resulted in inferior levels of produced extracellular enzyme. However, the relatively low cost of mineral salt media makes these results interesting for a large scale production of CphE_al_.

The expression of *cphE *in suitable *E. coli *strains greatly improved the amount of extracellular CGPase produced. This increase in productivity is an important step towards the industrial-scale production of dipeptides from CGP.

## Competing interests

The authors declare that they have no competing interests.
